# Professional Dyeing Work Enriches Dye-decolorizing Bacteria in the Fingertip Microbiome

**DOI:** 10.1264/jsme2.ME26026

**Published:** 2026-06-11

**Authors:** Tsukasa Ito

**Affiliations:** 1 Department of Civil and Environmental Engineering, Gunma University, Kiryu, Gunma, Japan

**Keywords:** dye-decolorizing bacteria, hand microbiome, occupational exposure, pollutant-degrading bacteria

## Abstract

Individuals who regularly handle specific chemicals may harbor microorganisms capable of metabolizing those compounds. Such hands may therefore provide a practical route for obtaining functional microorganisms from easily accessible human-associated environments. In this study, we found that nearly 90% of professional dyers harbored dye-decolorizing bacteria, which were efficiently isolated from their fingertips, with 20% of isolates exhibiting decolorizing activity. These strains showed distinct genus-level profiles. These findings indicate that occupational exposure to dyes strongly enriches dye-decolorizing bacteria, suggesting that the hands of textile workers may serve as a source for isolating microorganisms with potential applications in dye bioremediation.

Human hands harbor a diverse microbial community (Grice *et al.*, 2009; Edmonds-Wilson *et al.*, 2015) and show low interpersonal similarity (Grice *et al.*, 2009), making their community structure nearly as unique as fingerprints. Despite this variability, several bacterial families are consistently detected, suggesting shared functional traits. The human skin microbiota is shaped by diverse extrinsic and intrinsic factors (Boxberger *et al.*, 2021), and in particular, occupational exposure to microbial or chemical agents can influence its composition (Lai and Christiani, 2019).

We considered whether human hands could serve as a practical source for isolating pollutant-degrading bacteria, analogous to how oil-degrading microorganisms are obtained from contaminated environments (Das and Chandran, 2011). Although human hands are not continuously exposed to pollutants, as occurs in contaminated environments, repeated occupational exposure may still select for microorganisms capable of degrading these compounds. If hands function as an accessible reservoir of culturable pollutant-degrading microorganisms, they may provide an alternative to sampling from contaminated sites. Several skin-associated bacteria can reduce azo dyes used in cosmetics (Stingley *et al.*, 2010) and textile wastewater (Ito *et al.*, 2018). We therefore hypothesized that individuals who regularly handle specific chemicals may harbor microorganisms capable of metabolizing those compounds. To test this idea, we focused on the fingertips of professional dyers as a potential source of dye-decolorizing bacteria.

Fingertip-associated bacteria were collected from 111 participants, including 15 professional dyers with long-term occupational exposure to textile dyes. Each subject pressed six fingertips onto LB agar plates, which were incubated at 22°C for 3–7 days. From each participant, 10–20 colonies were randomly selected and purified, yielding 968 isolates. Dye-decolorizing activity was assessed using LB soft agar containing 20 mg L^–1^ Congo Red, following established procedures for evaluating azo-dye decolorization by skin-associated bacteria (Ito, 2013; Yamanashi and Ito, 2022). Each isolate was grown in LB and transferred into soft agar tubes incubated at 37°C before dye addition. Decolorization was evaluated visually after 48 h by comparison with reference tubes representing graded decolorization levels. Isolates were classified as dye-decolorizing strains when decolorization was judged by a near-complete loss of visible coloration, corresponding to a visually defined decolorization level exceeding 90%.

For community profiling, pooled dye-decolorizing and non-decolorizing isolates from each group were subjected to 16S rRNA gene sequencing on the MiSeq platform, and taxonomic assignment was conducted using QIIME2 with the SILVA database. Group differences were evaluated using non-parametric tests.

Phylogenetic anal­yses based on MiSeq sequencing data were performed to examine the taxonomic placement of dye-decolorizing isolates obtained from professional dyers. Neighbor-joining trees based on short nucleotide sequence data were constructed using the Maximum Composite Likelihood method in MEGA version 12 (Kumar *et al.*, 2024).

Prevalence of dye-decolorizing bacteria differed markedly among subject groups. Nearly all professional Dyers (93%) harbored at least one dye-decolorizing strain on their fingertips, whereas only 20–40% of Seniors, Adults, and Children carried such strains (Fig. 1A). Many individuals in the non-dyer groups lacked any dye-decolorizing isolates. Among all isolates obtained, approximately 20% of strains from Dyers exhibited dye-decolorizing activity, compared with less than 10% in the other groups (Fig. 1B). This calculation assumes random sampling of isolates and independence among sampling events. Based on this proportion, the probability of obtaining at least one dye-decolorizing strain when randomly sampling 10 isolates from Dyers was estimated to be ~88%. This value was calculated as 1–(1–*P*)^10^ with *P*=0.1934. This high probability indicates that dye-decolorizing bacteria are substantially more common on the fingertips of Dyers than on those of other individuals. These patterns suggest that long-term occupational exposure to dyes strongly influences the functional composition of fingertip-associated bacteria, overriding the typically high interpersonal variability reported for hand microbiota.

Repeated sampling of four Dyers over a one-year interval further supported the stability of this functional phenotype. All four individuals, each with more than 50 years of occupational exposure, consistently harbored dye-decolorizing bacteria, and the relative abundance of such isolates remained similar across sampling times (21→29%, 11→16%, 10→10%, and 9→10%). Although we did not determine whether the isolates represented identical strains across time, the recurrent detection of dye-decolorizing activity suggests that the fingertip environment of long-term Dyers supports the repeated presence or re-acquisition of such functional bacteria.

Persistent detection of functional phenotypes on skin has been reported in other contexts, including azo-dye reduction by skin bacteria (Stingley *et al.*, 2010) and the longitudinal stability of skin microbial communities (Oh *et al.*, 2016). This study focuses on detecting the functional enrichment of dye-decolorizing bacteria rather than determining whether these organisms are stably colonizing the skin. Our observations are consistent with the idea that chronic occupational exposure maintains dye-decolorizing capability at the individual level; however, the underlying mechanism—stable colonization versus continual transfer—cannot be determined in this study. Future studies using longitudinal sampling combined with strain-level genomic approaches, including whole-genome sequencing and multilocus sequence typing, will be required to distinguish stable colonization from repeated environmental transfer.

A preliminary single-subject trial in which three fingers were exposed to dye for 2–3 weeks did not show consistent increases in dye-decolorizing isolates relative to those from unexposed fingers. These observations suggest that short-term exposure may be insufficient to alter the fingertip microbiota, reinforcing the importance of long-term occupational exposure.

Hierarchical clustering of pooled bacterial communities revealed a clear separation between dye-decolorizing and non-decolorizing groups (Fig. 2). Communities from Children and Adults showed high similarity within each category, whereas the dye-decolorizing community from Dyers formed the most distinct branch. This reflected its‍ ‍unique genus-level profile, characterized by elevated proportions of *Kocuria*, *Exiguobacterium*, and *Neisseria* and‍ ‍a comparatively reduced share of *Bacillus*. The Senior dye-decolorizing community also showed partial separation from the younger groups, suggesting modest differentiation associated with age or exposure history.

Several genera enriched in the Dyers’ community include species known to reduce azo dyes (Saratale *et al.*, 2011; Uppala *et al.*, 2019; Singh and Bhadauria, 2023). *Kocuria*, *Exiguobacterium*, *Pseudomonas*, and *Bacillus* contain strains capable of degrading methyl red, Amido Black 10B,‍ ‍and related dyes. *Staphylococcus* species, including *Staphylococcus epidermidis* and *S. aureus*, also metabolize a variety of azo dyes through azoreductase activity. Although azo-dye decolorization has not previously been reported for the genus *Neisseria*, we obtained dye-decolorizing isolates belonging to this genus exclusively from professional dyers. Phylogenetic anal­ysis based on short sequence data confirmed that these isolates cluster within the *Neisseria* lineage, specifically within the subflava–mucosa–sicca group (Fig. 3A). In addition, dye-decolorizing isolates of *Exiguobacterium* obtained from dyers were phylogenetically distinct from previously reported decolorizing strains (*e.g.*, RD3, NIU-K4, and NIU-K2), indicating that dye-reducing capability can be present in additional lineages within this genus (Fig. 3B). Taken together with the known dye-reducing capabilities of other enriched genera, these findings indicate that the fingertips of professional dyers represent a source of both known and previously unrecognized dye-decolorizing bacteria.

The presence of azoreductase-like genes alone does not guarantee dye-decolorizing activity. Azo-dye reduction is a multifactorial process involving diverse oxidoreductases, electron-transfer pathways, and cellular redox states, and the efficiency of dye decolorization depends on the metabolic context of the whole cell rather than the presence of a single gene. The strong dye-decolorizing activity observed in our isolates therefore reflects a functional phenotype that emerges from integrated cellular physiology under the assay conditions. Long-term occupational exposure may favor strains in which these reductive pathways are physiologically active and ecologically advantageous in dye-exposed fingertip environments.

From a methodological perspective, the following points are noted. LB medium was used because it has been effective for isolating and evaluating dye-decolorizing skin bacteria (Ito, 2013; Ito *et al.*, 2018). Congo Red was used as a representative azo dye commonly employed in screening assays. Although the specific dyes handled by professional dyers vary, they predominantly belong to azo dye classes used for natural fibers, and dye-degrading bacteria are often capable of reducing multiple structurally related azo dyes. Accordingly, Congo Red is widely used as a model compound for evaluating azo-reductive activity (Stingley *et al.*, 2010; Ito *et al.*, 2018; Singh and Bhadauria, 2023).

In addition, limitations of the present study should also be acknowledged. The pooled isolate composition anal­ysis provided an overview of genus-level profiles, but cannot resolve individual-level variation or in situ relative abundance. Furthermore, the sample size of professional dyers was restricted by the availability of individuals with long-term occupational exposure, and broader sampling across dye-handling professions will be necessary to examine the generality of these patterns.

Finally, the use of human-derived isolates requires appropriate biosafety consideration, including evaluation of potential pathogenicity before any future application. Occupational chemical exposure also raises ethical and occupational-health questions regarding its influence on the human microbiome.

These findings have practical implications for isolating bacteria from human-associated environments. Sampling only a few Dyers may be sufficient to obtain dye-decolorizing strains, and sampling more workers could increase isolate diversity. Occupational exposure shapes skin microbial communities (Lai and Christiani, 2019), and repeated contact with specific chemicals may allow hands to‍ ‍serve as accessible reservoirs of pollutant-degrading microorganisms. Therefore, fingertip-based isolation may facilitate the discovery of additional dye-degrading microorganisms, including previously unrecognized functional groups. In this context, the fingertips of textile workers represent a promising source for isolating microorganisms relevant to dye degradation and water quality management, and may accelerate the discovery of strains with potential applications in bioremediation.

## Sequence data

Sequence data have been deposited in DDBJ under BioProject ID PRJDB40070.

## Declaration of Competing Interests

The author declares that there are no competing interests.

## Acknowledgements

I thank Siti Sarah Binti Abd Rahim, Sayako Shoda, Katsuyuki Okabe, and Yu Yamanashi for their contributions to data acquisition, including conducting experiments and collecting samples from volunteers. I thank Dr. Ikuo Tsushima for conducting the sequencing anal­ysis. I received assistance from Copilot for English editing of this manuscript. I also thank all volunteer participants who kindly provided bacterial samples from their fingerprints.

Funding: This work was supported by JSPS KAKENHI (grant number 15K12369).

### Data Availability

Data that support the findings of this study are available from the corresponding author, T. I., upon reasonable request.

## Citation

Ito, T. (2026) Professional Dyeing Work Enriches Dye-decolorizing Bacteria in the Fingertip Microbiome. *Microbes Environ ***41**: ME26026.

https://doi.org/10.1264/jsme2.ME26026

## Supplementary Material

Supplementary Material

## Figures and Tables

**Fig. 1.**
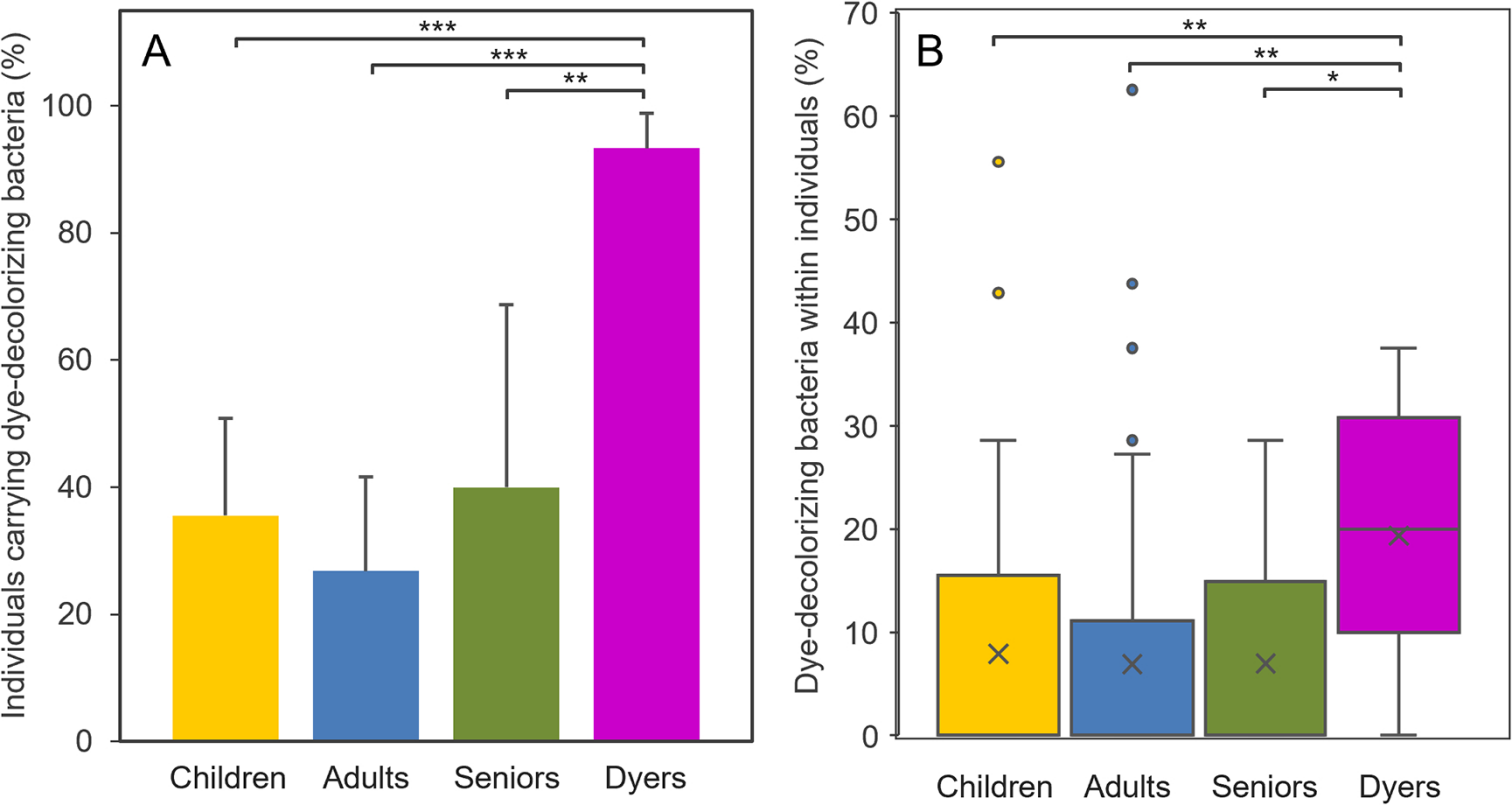
Distribution and prevalence of dye-decolorizing bacteria isolated from the fingertips of Children, Adults, Seniors, and professional dyers (Dyers). (A) Percentage of individuals carrying dye-decolorizing bacteria in each group. Error bars represent 95% confidence intervals (Wilson method). The Dyers group showed significantly higher carriage rates than the other groups (*, *P*<0.05; **, *P*<0.01). (B) Boxplots show the percentage of dye-decolorizing bacteria within each individual. Group sizes were Children (*n*=45), Adults (*n*=41), Seniors (*n*=10), and Dyers (*n*=15). The Dyers group exhibited significantly higher values than the other groups (**, *P*<0.01; ***, *P*<0.001). Pairwise comparisons among Children, Adults, and Seniors showed no significant differences.

**Fig. 2.**
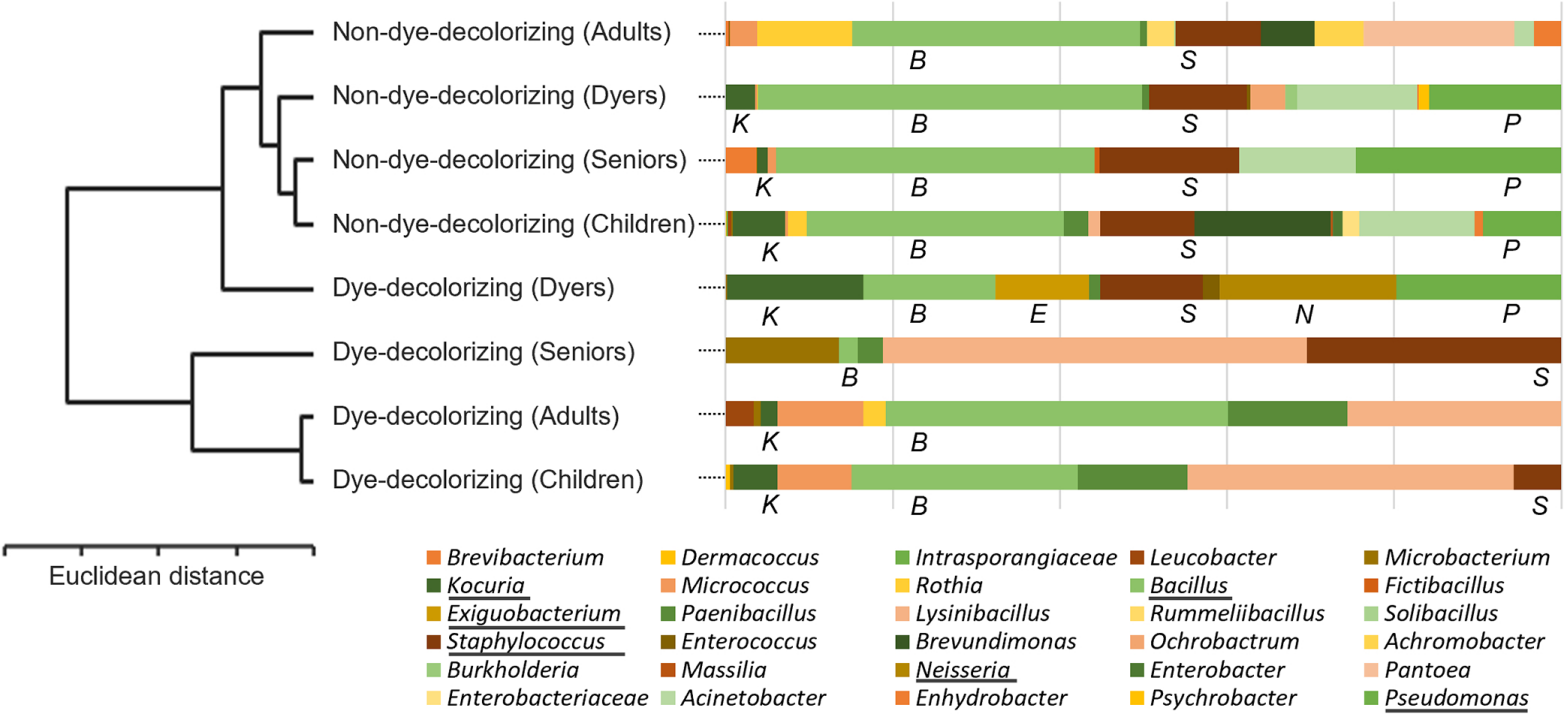
Hierarchical clustering and genus-level composition of fingertip bacterial communities isolated from dye-decolorizing and non-dye-decolorizing colonies. Eight pooled bacterial communities were generated from Children, Adults, Seniors, and professional dyers, separately for dye decolorizing and non-dye-decolorizing colonies. The dendrogram (left) shows the similarity among communities based on the Euclidean distance with Ward’s method. The stacked bar charts (right) represent the relative abundance of bacterial genera isolated from each community, with colors representing different genera. Distinctive genera are indicated below each bar by the initial letter of their genus names and are underlined in the legend.

**Fig. 3.**
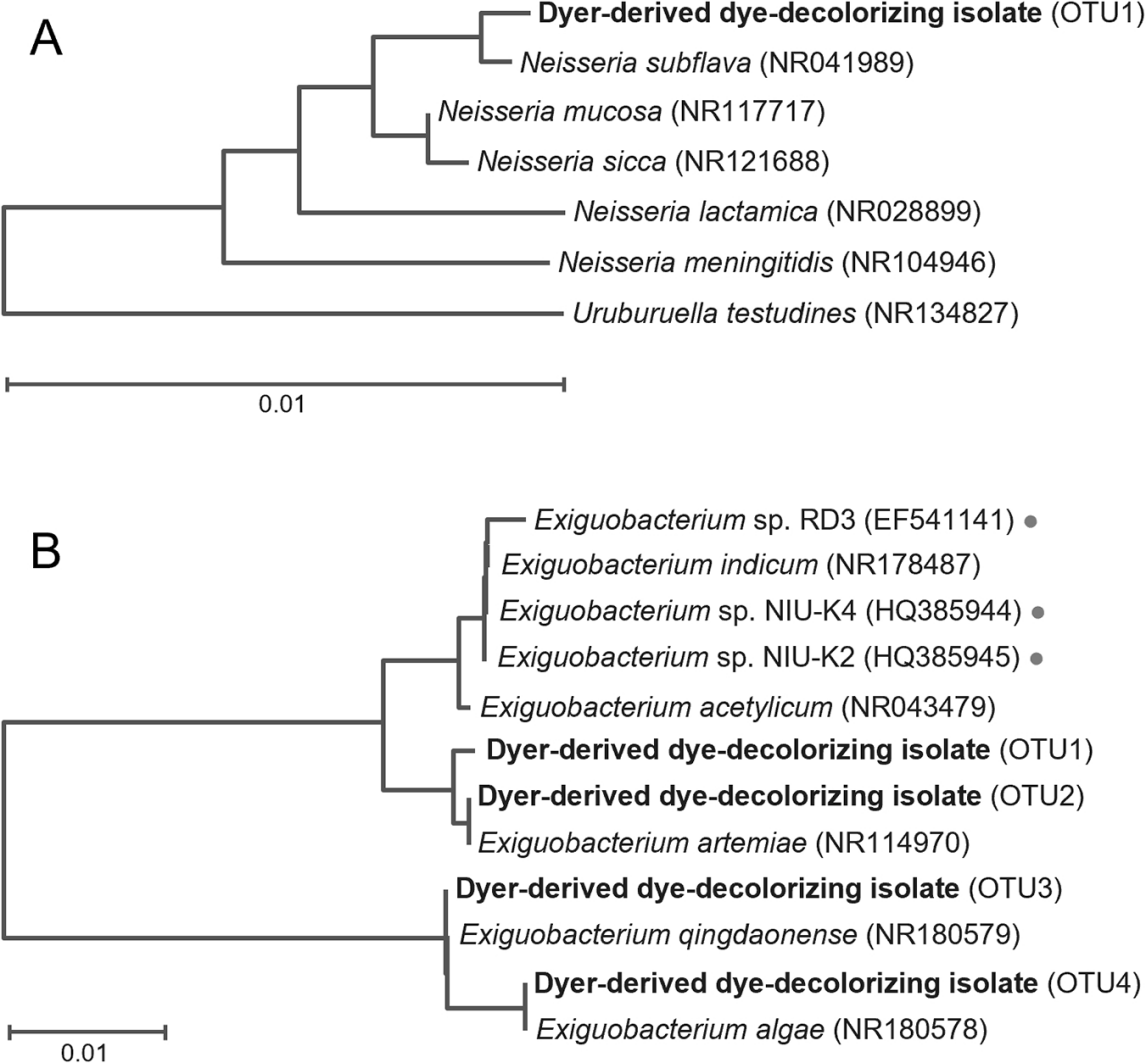
Phylogenetic placement of dye-decolorizing isolates obtained from professional dyers. Neighbor-joining trees based on short sequence data (417 bp) are shown for (A) *Neisseria* and (B) *Exiguobacterium*. Dyer-derived dye-decolorizing isolates are indicated. Previously reported dye-decolorizing strains of *Exiguobacterium* (RD3, NIU-K4, and NIU-K2) are indicated by closed circles (●). *Uruburuella testudines* was used as an outgroup in the *Neisseria* tree.
