# Supplementary material for: Professional Dyeing Work Enriches Dye-decolorizing Bacteria in the Fingertip Microbiome
Source: Microbes Environ. 2026 Jun 11;41(2):ME26026. doi: 10.1264/jsme2.ME26026 (PMC13293701; doi:10.1264/jsme2.ME26026)
Supplement: Supplementary file 1 — Supplementary Material [file 41_26026_s1.pdf]

Microbes Environ. 41(2), 2026
https://www.jstage.jst.go.jp/browse/jsme2 doi:10.1264/jsme2.ME26026

Supplementary Materials

Professional Dyeing Work Enriches Dye-Decolorizing Bacteria in the Fingertip Microbiome

Tsukasa Ito[1]*

[1]*Department of Civil and Environmental Engineering, Gunma University, Kiryu, Gunma, Japan*

*Corresponding author. E-mail: t.ito@gunma-u.ac.jp

Materials and Methods

Study population and sampling design

A total of 111 human subjects aged 3–88 years participated in this study. The cohort consisted of 15 professional dyers (39–85 years old), 45 children (3–11 years), 41 adults (20–58 years), and 10 seniors (> 60 years). All dyers, except

one individual with 10 years of experience, had more than 30 years of occupational exposure to textile dyes.

To collect fingertip-associated bacteria, each participant gently pressed the fingertips of three fingers (thumb, index, and middle finger) of one hand onto an LB agar plate, and repeated the procedure with the other hand, yielding samples from six fingertips per person. Plates were incubated aerobically at room temperature (22 °C) for 3–7 days depending on colony density. From each participant, 10–20 colonies were randomly selected without using group identity as a criterion and purified on LB agar. Because colony morphology does not allow identification of dye-decolorizing strains, no intentional selection was possible. Media-only plates confirmed that no colonies appeared in the absence of inoculum. In total, 355 isolates from children, 331 from adults, 81 from seniors, and 201 from dyers were obtained and subsequently tested for dye-decolorizing activity.

Assay for dye-decolorizing activity

Dye-decolorizing ability was assessed using LB soft agar (0.3%) containing Congo Red at a final concentration of 20 mg/L, following our previous studies (Ito 2013; Yamanashi & Ito 2022) with modifications. The use of soft agar enabled visualization of decolorization under different oxygen conditions: anaerobic strains decolorized dye except near oxygen-exposed regions, whereas aerobic strains decolorized from the gas–liquid interface downward.

Each purified colony was first inoculated into 1.2 mL LB liquid medium in a 1.5 mL tube. A 0.3 mL aliquot was transferred into each of three test tubes

containing 5 mL LB soft agar. Tubes were incubated at 37 °C for 17 h to ensure sufficient biomass before dye addition while avoiding entry into stationary phase. Congo Red solution (100 µL of 1 g/L stock) was then added to achieve a final concentration of 20 mg/L. Tubes were incubated at 37 °C for 48 h, and decolorization was visually assessed.

Dye concentrations were estimated using a colorimetric comparison with standard tubes containing 0–20 mg/L Congo Red (corresponding to 100–0% remaining dye). Strains achieving ≥90% decolorization were classified as dye-decolorizing bacteria. This non-contact measurement method enabled rapid evaluation of dozens of samples simultaneously without removing liquid from the tubes.

Preparation of pooled isolate compositions and 16S rRNA gene sequencing

All isolates from each subject group were combined separately for dye-decolorizing and non-dye-decolorizing strains These pooled samples represent the taxonomic composition of cultured isolates and do not reflect in situ skin microbiomes or relative abundance. DNA was extracted from each pooled isolate compositions using the DNeasy Blood & Tissue Kit (Qiagen, Germany). PCR amplification of the 16S rRNA gene was performed using the universal primer pair Pro341F/Pro805R (Takahashi et al., 2014). Sequencing libraries were prepared following the Illumina 16S Metagenomic Sequencing Library Preparation Guide (Illumina, San Diego, USA). Sequencing was conducted on the MiSeq platform using the MiSeq Reagent Kit v3 (2 × 300 bp configuration). Raw sequence data were demultiplexed based on barcode information, and primer sequences were removed. Low-quality reads were

discarded, paired-end reads were merged, and chimera and noise filtering were performed, resulting in approximately 80,000 high-quality reads per sample. Taxonomic assignment was conducted using QIIME2 (Bolyen et al., 2019) with taxonomic assignment against the SILVA 132 database (Quast et al., 2013). Genus-level profiles of isolated strains were used for hierarchical clustering analysis with Mulcel2 software (OMS Publishing Inc., Japan).

Phylogenetic analyses of dyer-derived dye-decolorizing isolates

Phylogenetic analyses were performed to examine the taxonomic placement of dye-decolorizing isolates obtained from professional dyers. Neighbor-joining trees were constructed based on short nucleotide sequence data using the Maximum Composite Likelihood method to calculate evolutionary distances. Ambiguous positions were removed using the pairwise deletion option, resulting in a final alignment of 417 positions. All phylogenetic analyses were conducted using MEGA version 12 (Kumar et al., 2024).

Statistical analysis

Group differences in the percentage of dye-decolorizing bacteria were assessed using the Kruskal–Wallis test ($H = 18.04$, $df = 3$, $p < 0.001$), followed by Steel–Dwass multiple comparisons. Differences in the proportion of individuals carrying dye-decolorizing bacteria were evaluated using Fisher's exact tests and chi-square tests ($\chi^2 = 21.01$, $df = 3$, $p < 0.001$). Effect sizes for categorical comparisons were quantified using Cramér's V ($V = 0.435$). Confidence intervals for proportions were calculated using the Wilson method. All statistical analyses were performed using standard non-parametric procedures appropriate for non-normally distributed data.

References

Bolyen, E., Rideout, J.R., Dillon, M.R., Bokulich, N.A., Abnet, C.C., Al-Ghalith, G.A. *et al.* (2019) Reproducible, interactive, scalable and extensible microbiome data science using QIIME 2. *Nat Biotechnol* **37**: 852–857.

Kumar, S., Stecher, G., Suleski, M., Sanderford, M., Sharma, S., and Tamura, K. (2024) MEGA 12: Molecular Evolutionary Genetics Analysis version 12 for adaptive and green computing. *Mol Biol Evol* **41**: 1–9.

Ito, T. (2013) Color-removal by microorganisms isolated from human hands. *J Microbiol Biol Educ* **14**: 244–247.

Quast, C., Pruesse, E., Yilmaz, P., Gerken, J., Schweer, T., Yarza, P. *et al.* (2013) The SILVA ribosomal RNA gene database project: improved data processing and web-based tools. *Nucleic Acids Res* **41**: D590–D596.

Takahashi, S., Tomita, J., Nishioka, K., Hisada, T., and Nishijima, M. (2014) Development of a prokaryotic universal primer for simultaneous analysis of Bacteria and Archaea using next-generation sequencing. *PLoS One* **9**: e105592.

Yamanashi, Y., and Ito, T. (2022) A minority population of non-dye-decolorizing *Bacillus subtilis* enhances the azo dye-decolorizing activity of *Enterococcus faecalis*. *Microbes Environ* **37**: ME21080.
